# Metabolic syndrome and its components are associated with lengths of stay in a psychiatric hospital: Results from a Swiss psychiatric cohort and first-episode psychosis patients

**DOI:** 10.1192/j.eurpsy.2025.10036

**Published:** 2025-05-26

**Authors:** Nermine Laaboub, Setareh Ranjbar, Séverine Crettol, Nicolas Ansermot, Frederik Vandenberghe, Carole Grandjean, Marianna Piras, Julien Elowe, Martin Preisig, Armin Von Gunten, Philippe Conus, Chin Bin Eap

**Affiliations:** 1 Unit of Pharmacogenetics and Clinical Psychopharmacology, Centre for Psychiatric Neuroscience, Department of Psychiatry, Lausanne University Hospital, University of Lausanne, Prilly, Switzerland; 2 Center for Psychiatric Epidemiology and Psychopathology, Department of Psychiatry, Lausanne University Hospital, University of Lausanne, Prilly, Switzerland; 3 Service of Adult Psychiatry North-West, Department of Psychiatry, Lausanne University Hospital, University of Lausanne, Prilly, Switzerland; 4 Service of Old Age Psychiatry, Department of Psychiatry, Lausanne University Hospital, University of Lausanne, Prilly, Switzerland; 5 Service of General Psychiatry, Department of Psychiatry, Lausanne University Hospital, University of Lausanne, Prilly, Switzerland; 6 Les Toises Psychiatry and Psychotherapy Center, Lausanne, Switzerland; 7 University of Lausanne, Lausanne, Switzerland

**Keywords:** lengths of stay, metabolic disturbances, metabolic syndrome, psychiatry

## Abstract

**Background:**

Due to limited inpatient care resources and high healthcare expenditures, understanding factors that affect lengths of stay (LOS) is highly relevant. We aimed to investigate associations between metabolic disturbances and LOS in a psychiatric hospital and to identify other clinical and sociodemographic LOS predictors.

**Methods:**

Patients admitted to one of the units of the general psychiatric or psychogeriatric departments between January 1, 2007 and December 31, 2020, were included. Metabolic disturbances (i.e., the metabolic syndrome and its five components) were defined using the International Diabetes Federation definition. Cox frailty regression models with time-varying coefficients were used to investigate the association between metabolic disturbances and LOS. Hazard ratios (HR) >1 and HR < 1 indicated the relative likelihood of shorter and extended LOS, respectively.

**Results:**

A total of 7,771 patients for 16,959 hospital stays throughout 14 years of follow-up were included. Central obesity (HR = 0.82; 95% confidence interval [CI] = [0.76–0.89]), hyperglycemia (HR = 0.83; 95% CI = [0.78–0.89]), hypertriglyceridemia (HR = 0.87; 95% CI = [0.80–0.92]), and the metabolic syndrome (HR = 0.76; 95% CI = [0.70–0.82]) were associated with an increased risk of extended LOS in the psychiatric hospital, while underweight (HR = 1.30, 95% CI = [1.09–1.56]) and HDL hypocholesterolemia (HR = 1.10, 95% CI = [1.03–1.18]) were associated with a higher likelihood of shorter LOS. In first-episode psychosis patients, hypertriglyceridemia (HR = 0.82; 95% CI = [0.67–0.99]) and hypertension (HR = 0.76, 95% CI = [0.58–0.99]) were associated with extended LOS when considering all stays, while no association was found when considering the first stay per patient.

**Conclusion:**

Future studies should determine whether better metabolic monitoring and treatment of metabolic disturbances can contribute to reducing LOS.

## Introduction

Psychiatric patients are often admitted to psychiatric facilities, requiring multidisciplinary management including psychiatrists, nurses, psychologists, and social workers [1]; the necessity of it may lead to extended lengths of stay (LOS) and high healthcare expenditures [2, 3]. In the context of incentives for health authorities, insurers, and care providers to improve patient care while simultaneously lowering healthcare costs [4, 5], achieving both is challenging, with one path being to reduce LOS [6]. Many studies have been conducted on the economic and clinical benefits of reducing LOS in psychiatry. Some studies concluded that extended LOS do not appear to reduce psychiatric readmission rates or improve other clinical outcomes [7, 8], while others suggested that premature discharge, particularly for patients with severe disorders, can lead to a high risk of relapse and readmission, raising healthcare costs [4]. Besides, from the patients’ perspective, long LOS may be unpleasant, isolating, stigmatizing, and cause a loss of autonomy [7, 9].

Epidemiological studies examining patient-level characteristics revealed that smoking [4], being married, being detained, and either young or middle-aged are all associated with shorter LOS [10]. Conversely, older age, male sex, White ethnicity, or homelessness are all associated with extended LOS [6, 10]. In addition, LOS are influenced by the illness’s level of complexity (notably psychotic disorders) [10], and treatment compliance or resistance [11]. Furthermore, according to a meta-analysis, 55% of those who experienced their First Episode of Psychosis (FEP) required hospital stays during a mean follow-up of 7 years subsequent to the first episode, and 34% met the criteria for treatment resistance, resulting in a pooled average LOS of 116 days [12]. Likewise, psychiatric patients are predisposed to metabolic disturbances as a result of psychotropic treatments (for most antipsychotics and mood stabilizers, and some antidepressants), lifestyle, medical conditions (e.g., insomnia disorders), and/or genetics [13–18]. We recently reported that metabolic disturbances, namely high Body Mass Index (BMI), central obesity, hyperglycemia, and metabolic syndrome, are associated with psychiatric readmission in two cohorts (PsyMetab and PsyClin). Moreover, obesity and High-Density Lipoprotein (HDL) hypercholesterolemia doubled the risk of readmission in a cohort of FEP patients [19]. Nevertheless, in the abovementioned psychiatric cohorts, the relationship between LOS and metabolic disturbances was not studied.

The present study aimed to (1) investigate the associations of metabolic disturbances, namely high BMI, central obesity, hyperglycemia, hypertriglyceridemia, HDL hypocholesterolemia, hypertension, and the metabolic syndrome, with LOS, in a Swiss psychiatric hospital; (2) identify other clinical and sociodemographic LOS predictors; and (3) replicate the findings in a subgroup of FEP patients.

## Methods

### Study design, setting, and participants

This study used data from PsyMetab and PsyClin, two prospective cohort studies carried out in the Department of Psychiatry of the University Hospital of Lausanne in Switzerland. Detailed methods have been previously published [16]. Briefly, patients who were starting or continuing psychotropic treatment with a potential risk of inducing metabolic disorders (listed in Supplementary Table 1) were recruited. In addition, a subgroup of FEP patients (i.e., patients with the shortest duration of illness of the whole cohort and no or minimal history of prescription of psychotropic drugs [no longer than 6 months]) were recruited from the Treatment and Early Intervention in Psychosis Program (TIPP) [20] and the PsyMetab and PsyClin studies. This research included inpatients from general psychiatry or psychogeriatric departments between January 1, 2007 and December 31, 2020.

### Ethical approval

Patients who have given their informed consent for the PsyMetab study (CER-VD = 2017-01301) or those accepting the general consent of the Lausanne University Hospital have been included. In addition, due to the non-interventional post-hoc analysis study design, the Ethics Committee of the Canton of Vaud (CER-VD) has approved the use of clinical data from patients followed between 2007 and 2015 without informed consent (PsyClin; CER-VD = 2016-00281). Patients included in the TIPP cohort gave their written informed consent, and the TIPP protocol was approved by CER-VD (2020-00272).

### Data extraction

LOS predictors were selected based on the existing literature and the availability of the data in patients’ electronic files.

- The sociodemographic information collected included age, sex, marital status, education, living situation, and employment. To calculate the Swiss socioeconomic position (range: 0 [most disadvantaged] to 100 [most privileged]), patient addresses were extracted and geocoded using the Google API and the ggmap R package [21, 22].
- Clinical characteristics at admission were recorded, namely admission status (voluntary or compulsory), the clinical severity at admission assessed using the Health of the Nation Outcome Scales (HoNOS; an instrument comprising 12 simple scales measuring behavior, impairment, symptoms, and social functioning; the total score ranges from 0 [no problems] to 48 [severe problems]), psychiatric diagnoses based on ICD-10 codes classified into seven diagnostic groups as follows: dementia (F00–F02 and G30), intellectual disabilities (F70–F79), substance use disorders (F10–F19), psychotic disorders (F20–F25 and F28–F29), bipolar disorders (F30–F31), depression (F32–F33), and other diagnoses (F03–F09, F34–F69, and F80–F99; used as reference category in models), and psychotropic medications characterized by risk of weight gain (Supplementary Table 1). Smoking status was established based on self-reported tobacco consumption, the presence of ICD-10 mental and behavioral disorders due to the use of tobacco, and/or the use of nicotine or varenicline.
- Metabolic disturbances were defined as follows: BMI was calculated as weight in kilograms divided by height in square meters. Following the International Diabetes Federation definition, laboratory values and ATC codes were used to determine the presence of the metabolic syndrome and/or its five components (central obesity, hyperglycemia, hypertriglyceridemia, HDL hypocholesterolemia, and hypertension; Supplementary Tables 2A,B) [23].

### Statistical analyses

The cohort characteristics are presented as numbers and percentages, or medians and interquartile ranges (IQR), for categorical or continuous variables, respectively. Univariate analyses were performed using Cox frailty regression models to identify separately predictors of LOS and investigate the eventual time-dependence of their effect (proportionality assumption) [24, 25]. Subsequently, a multivariable model was performed to investigate the effect of the metabolic syndrome on LOS, accounting for clinical and demographic covariates with a *p*-value ≤ 0.1 in univariate analyses and <60% of missing values (to ensure a sufficient sample size). As the clinical covariates, but not our exposure (i.e., the metabolic disturbances), showed a time-varying effect on LOS in univariate tests, the model was fitted by including only the average effect of those covariates. In addition, a sensitivity analysis was conducted to account for the time-varying effects of the clinical covariates. The model was then replicated by substituting each metabolic disturbance for the metabolic syndrome variable, resulting in a total of seven models, since we cannot include all in one model due to multicollinearity. The outcomes of the multivariable models are presented as hazard ratios (HR). An HR > 1 reflected a higher likelihood of a shorter LOS compared to the median, whereas an HR < 1 indicated a higher likelihood of a longer LOS than the median. In addition, subgroup analyses using Cox frailty regression models (considering and ignoring the time-varying effects, respectively) and Cox regression models were conducted in FEP patients by considering all hospital stays during the follow-up and the first hospital stay by patient, respectively. Data preparation was done using STATA version 16.0 for Windows (StataCorp, TX, USA), and univariate and multivariate analyses were performed using R version 4.2.2 for Windows (GNU General Public License version 2, MA, USA). All the presented results are statistically significant at the 95% level.

## Results

### Cohort characteristics

A total of 7,771 patients for 16,959 hospital stays throughout 14 years of follow-up were included in the present study. The median LOS was 23 days (IQR: 11–45 days), while the mean was 39 days (standard deviation [SD]: 54 days). Supplementary Table 3 displays the mean, SD, median, and first and third quartiles of LOS by year.

Table 1 shows the sociodemographic and clinical characteristics of the cohort. The median age was 44 years, with the majority being female (53%). Patients were more frequently unemployed or on pensions, with a median socioeconomic position of 56. In terms of clinical characteristics, the predominant psychiatric disorders were psychotic, depressive, and substance use disorders; more than half the patients were admitted voluntarily (56%), and the median HoNOS was 20. Psychotropic medications were considered in terms of their potential to induce weight gain, with 23% of patients using those known for a risk of weight gain.

**Table 1.** Sociodemographic and clinical characteristics of patients

Characteristics	Total of admissions *N* = 16,959	Characteristics	Total of admissions *N* = 16,959
LOS, median (IQR), days	23 (11–45)	HoNOS score at admission, median (IQR)	20 (14–26)
Age, median (IQR), years	44 (32–60)	SSEP median (IQR)	56 (45–65)
Sex, *n* (%) Men Women	8,023 (47) 8,936 (53)	Smokers, *n* (%) No Yes	2,850 (39) 4,417 (61)
Marital status, *n* (%) Married or registered partnership Single Divorced or separated Widowed	3,104 (18) 8,155 (48) 4,214 (25) 1,419 (9)	Education, *n* (%) Compulsory schooling No schooling Apprenticeship High school University or college	573 (27) 164 (8) 697 (33) 138 (7) 516 (25)
Employment, *n* (%) Employed Unemployed Pensions (disability, retirement) Other (e.g., student)	371 (12) 741 (25) 1,823 (60) 97 (3)	Admission status, *n* (%) Voluntary Compulsory	1,951 (56) 1,510 (44)
Living situation, *n* (%) Home, with others Home, alone Homeless Institutions Others	1,304 (39) 1,086 (32) 117 (3) 703 (21) 160 (5)	Psychiatric disorders, *n* (%) Dementia Intellectual disabilities Substance use disorders Psychotic disorders Bipolar disorders Depressive disorders Other	1,356 (9) 361 (2) 3,040 (19) 5,934 (38) 1,857 (12) 3,032 (19) 217 (1)
Psychotropic treatments by risk of weight gain, *n* (%) No risk of weight gain Risk of weight gain	13,064 (77) 3,895 (23)		

*n* varies due to missing values.

Psychiatric diagnoses were defined as follows: dementia (F00–F02 and G30); intellectual disabilities (F70–F79); substance use disorders (F10–F19); psychotic disorders (F20–F25 and F28–F29); bipolar disorders (F30–F31); depression (F32–F33); and other diagnoses (F03–F09, F34–F69, and F80–F99).

Psychotropic treatments were defined as follows: Risk of weight gain = amisulpride, amitriptyline, aripiprazole, asenapine, brexpiprazole, carbamazepine, cariprazine, chlorprothixene, clomipramine, clotiapine, clozapine, doxepin, flupentixol, haloperidol, levomepromazine, lithium, lurasidone, mirtazapine, nortriptyline, olanzapine, opipramol, pipamperone, pregabalin, promazine, quetiapine, risperidone/paliperidone, sertindole, sulpiride, tiapride, trimipramine, valproate, and zuclopenthixol. No risk of weight gain = all the other psychotropic medications.

Abbreviations: HoNOS, Health of the Nation Outcome Scales; IQR, interquartile range; LOS, lengths of stay; *n*, number; SSEP, Swiss socioeconomic position.

Table 2 displays the metabolic characteristics of patients at admission. The median BMI was 25.2 kg/m^2^, with 30 and 22% of patients being overweight and obese, respectively. Almost one-third of the cohort had central obesity, hypertriglyceridemia, HDL hypocholesterolemia, and/or hyperglycemia. Finally, 15% of patients had metabolic syndrome.

**Table 2.** Metabolic characteristics of patients at admission

Characteristics	Total of admissions *N* = 16,959
Weight, median (IQR), kg	72 (61–84)
BMI, median (IQR), kg/m^2^	25.2 (22.0–29.4)
BMI categories, *n* (%) Normal (18.5 kg/m^2^ ≤ BMI < 25 kg/m^2^) Underweight (BMI < 18.5 kg/m^2^) Overweight (25 kg/m^2^ ≤ BMI < 30 kg/m^2^) Obese (BMI ≥ 30 kg/m^2^)	4,007 (43) 498 (5) 2,779 (30) 2,099 (22)
Waist circumference, median (IQR), cm Men Women	96 (86–109) 91 (80–104)
Central obesity, *n* (%), IDF definition Yes No	2,936 (31) 6,575 (69)
Plasma cholesterol Triglycerides, median (IQR), mmol/L HDL, median (IQR), mmol/L Men Women LDL, median (IQR), mmol/L Total, median (IQR), mmol/L	1.3 (0.9–1.8) 1.2 (1.0–1.5) 1.5 (1.2–1.8) 2.7 (2.1–3.4) 4.8 (4.1–5.6)
Hypertriglyceridemia, *n* (%) Yes No	3,094 (32) 6,573 (68)
HDL hypocholesterolemia, *n* (%) Yes No	3,280 (31) 7,182 (69)
Systolic blood pressure, median (IQR), mmHg	121 (110–135)
Diastolic blood pressure, median (IQR), mmHg	76 (68–85)
Hypertension, *n* (%) Yes No	3,684 (78) 1,019 (22)
Fasting plasma glucose, median (IQR), mmol/L	5.1 (4.7–5.8)
Hyperglycemia, *n* (%) Yes No	3,499 (29) 8,381 (71)
Metabolic syndrome, *n* (%), IDF definition Yes No	1,527 (15) 8,553 (85)

*n* varies due to missing values.

Abbreviations: BMI, body mass index; cm, centimeter; HDL, high-density lipoprotein; IDF, International Diabetes Federation; IQR, interquartile range; kg, kilogram; LDL, low-density lipoprotein; m, meter; mmHg, millimeters of mercury; mmol, millimole; *n*, number of observations.

### Metabolic and other factors associated with LOS in the whole cohort

**Figure 1.**
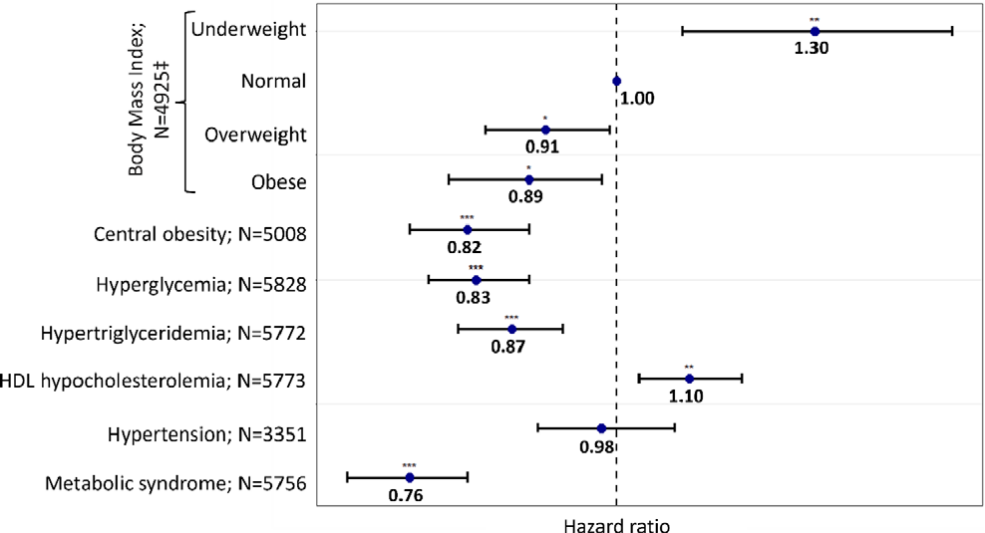
Association between metabolic disturbances and lengths of stay (LOS) in all cohorts. *N* varies due to missing values. *p-value < 0.05; **p-value < 0.01; ***p-value < 0.001. ‡Compared to normal weight and defined as follows: normal weight (reference): 18.5 ≤ body mass index (BMI) ≤ 25 kg.m^−2^; underweight: BMI < 18.5 kg.m^−2^; overweight: 25 kg.m^−2^ ≤ BMI ≤ 30 kg.m^−2^; obese: BMI > 30 kg.m^−2^. Metabolic disturbances were defined using the International Diabetes Federation definition. Each model was adjusted for age, sex, smoking status, psychiatric diagnoses, psychotropic medication, previous hospital admission, and year. Abbreviations: HDL, high-density lipoprotein; *N*, number of observations in each model.

The first Cox frailty model, which included the metabolic syndrome and accounted for variables linked to LOS according to the univariate Cox frailty models with a lenient significance level of *p* < 0.1, failed to meet the proportionality assumption for smoking, prior hospital admission, and psychiatric diagnosis variables. This suggests the time-varying effects of these factors on LOS. Nevertheless, the interaction terms with time were all at ~1 (data not shown). Disregarding the time-varying effects of these factors does not influence the association between our exposure (i.e., the metabolic disturbances) and LOS. Thus, the mean effect of those variables on LOS over time was considered by ignoring time interactions in the models. The association between LOS and the metabolic syndrome, adjusted for covariates and excluding time interactions (*N* = 5,756), is displayed in Figure 1 and Supplementary Table 4. Patients with a metabolic syndrome had a 24% (HR = 0.76) higher likelihood of experiencing extended LOS (95% confidence interval [CI] = [0.70–0.82]). In addition, advanced age (65 years and older) and a history of a prior admission to a psychiatric hospital were associated with a 40% (95% CI = [0.51–0.70]) and 8% (95% CI = [0.85–0.99]) increased risk of extended LOS, respectively (Supplementary Table 4), whereas LOS exhibited a decrease over time (HR = 1.04; 95% CI = [1.03–1.05]) (i.e., each additional year was associated with a 4% increased likelihood of shorter LOS). No significant relationship was observed between LOS, sex, and the use of weight-gain-inducing psychotropic drugs. Smoking and age between 25 and 39 years on the one hand and being diagnosed with dementia on the other tended to be linked to shorter and extended LOS, respectively.

Figure 1 displays the outcomes of Cox frailty models, wherein the metabolic syndrome was substituted by each metabolic disturbance to assess its potential as a predictor of LOS. Depending on the metabolic disturbance under consideration, the models accounted for variations in LOS ranging from 38% to 42%. Regarding BMI, being underweight was associated with a 30% increased likelihood of shorter LOS (95% CI = [1.09–1.56]), while being overweight or obese was associated with a 9% and 11% increased risk of extended LOS, respectively (95% CI = [0.84–0.99] and 95% CI = [0.80–0.98]). A 10% increased chance of shorter LOS was associated with HDL hypocholesterolemia (95% CI = [1.03–1.18]), whereas central obesity (HR = 0.82, 95% CI = [0.76–0.89]), hyperglycemia (HR = 0.83, 95% CI = [0.78–0.89]), and hypertriglyceridemia (HR = 0.87, 95% CI = [0.80–0.92]) were all associated with an increased likelihood of extended LOS. Hypertension did not exhibit a significant association with LOS (HR = 0.98, 95% CI = [0.90–1.08]).

### Sensitivity analyses in the whole cohort

Sensitivity analyses were performed by considering the time-varying effects of smoking, prior psychiatric hospital admission, and psychiatric diagnosis variables, in addition to the aforementioned factors. Smoking, intellectual disabilities, substance use disorders, depression, and a previous admission to a psychiatric hospital were all associated with a 15% (95% CI = [1.05–1.26]), 3.1-fold (95% CI = [1.50–6.20]), 2.6-fold (95% CI = [1.32–4.92]), 2.1-fold (95% CI = [1.11–4.13]), and 11% (95% CI = [1.01–1.22]) higher likelihood of shorter LOS, respectively (Supplementary Table 5). It is noteworthy that similar results were observed for the other factors, namely metabolic disturbances, regardless of whether the aforementioned interactions with time were considered or not (Supplementary Figure 1 and Supplementary Table 5).

### Metabolic factors associated with LOS in the FEP patients (subgroup analyses)

Supplementary Tables 6–8 display the baseline sociodemographic, clinical, and metabolic characteristics of the FEP patients (*N* = 1,134, number of patients = 289), as well as the distribution of LOS over years.

**Figure 2.**
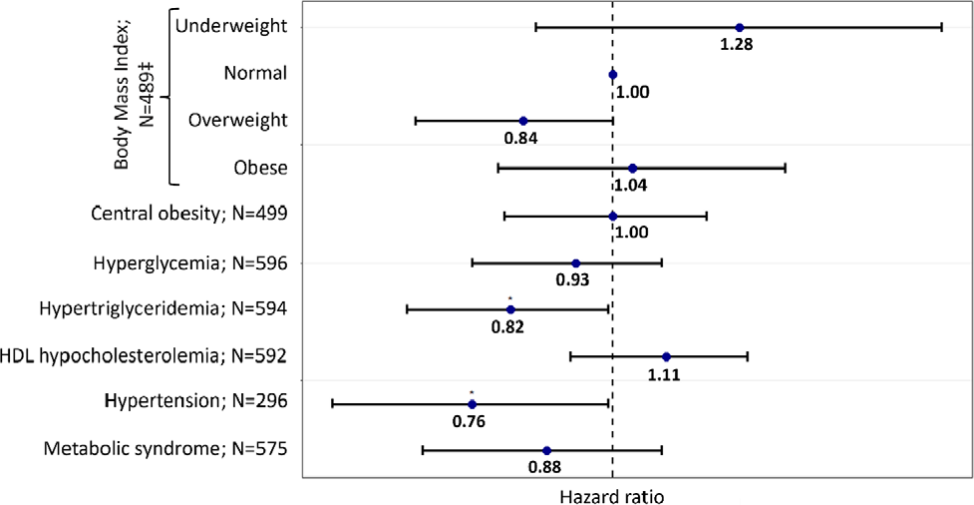
Association between metabolic disturbances and lengths of stay (LOS) in First-Episode Psychosis patients (all hospital stays): *N* varies due to missing values. *p-value < 0.05. ‡Compared to normal weight and defined according to the World Health Organization (WHO) definition: normal weight (reference): 18.5 kg.m^−2^ ≤ body mass index (BMI) ≤ 25 kg.m^−2^; Underweight: BMI < 18.5 kg.m^−2^; overweight: 25 kg.m^−2^ ≤ BMI ≤ 30 kg.m^−2^; Obese: BMI > 30 kg.m^−2^. Metabolic disturbances were defined using the International Diabetes Federation definition. Each model was adjusted for age, sex, smoking status, previous hospital admission, psychotropic medication, year, and interaction between time and previous hospital stay. Abbreviations: HDL, high-density lipoprotein; *N*, number of observations in the model.

Regarding multivariate analyses, as far as all hospital stays are considered, Cox frailty models failed to satisfy the proportionality assumption for the variable prior psychiatric hospital admission. However, regardless of whether this interaction with time was considered (Figure 2 and Supplementary Figure 2), hypertriglyceridemia (HR = 0.82, 95% CI = [0.67–0.99]; *N* = 594) and hypertension (HR ≤ 0.72, 95% CI = [0.58–0.99]; *N* = 296) were associated with an increased risk of having extended LOS. The relationship between the other metabolic disturbances and LOS did not reach the statistically significant threshold.

No significant association between LOS and metabolic disturbances was observed among FEP patients when considering only the first hospital stay per patient, except for hypertriglyceridemia, which tended to be associated with extended LOS (HR = 0.66; 95% CI = [0.41–1.06]; *p* = 0.09; *N* = 130; Supplementary Figure 3).

## Discussion

The present study is the first, to our knowledge, to identify metabolic disturbances as predictors of LOS in a psychiatric hospital across a 14-year follow-up period in a large cohort of psychiatric patients, including those experiencing an FEP. With the exception of HDL hypocholesterolemia, which was associated with shorter LOS, elevated BMI, metabolic syndrome, and its components were all associated with extended LOS when considering the whole cohort. In FEP patients, hypertriglyceridemia and hypertension were associated with extended LOS when considering all stays, while no association was found when considering the first stay per patient.

Our multivariable models explained 38%–42% of LOS variation, which is consistent with the findings of other studies [1, 6, 26, 27]. Of note, service-level factors and information pertaining to healthcare funding mechanisms were not considered in the present study. Because private healthcare insurance is mandatory in Switzerland [27], funding mechanisms are necessarily limited in contributing to the variance of LOS. A 59.6% variance in LOS was observed in a study (*n* = 97) that encompassed both patient and service-level factors [28]. Additional large studies considering patient-level factors (e.g., the metabolic profile), in addition to other factors (e.g., the healthcare funding mechanism and service-level factors), are needed to improve the explained LOS variance.

Several studies, with inconsistent findings, have investigated the relationship between somatic comorbidities and LOS in psychiatric hospitals. Some studies documented a positive association [29, 30], while others reported either no association or a weakly significant one [5, 31]. This is likely attributed to the applied definition of somatic comorbidities and the fact that some studies did not specify the physical comorbidity considered, limiting comparison. Moreover, no study has solely focused on metabolic disturbances.

Low BMI (underweight) was associated with shorter LOS than normal BMI, whereas high BMI (overweight and obese) was associated with extended LOS. At admission, low BMI was associated with extended LOS in adult females with anorexia nervosa [32], and a recent meta-analysis reported a pooled mean LOS of 76.3 days in such patients [33]. Since fewer than 1% of our patients had anorexia nervosa, no comparison could be made. A study revealed that patients with a high BMI (≥25 kg/m^2^) had delayed clinical response and reduced neuroendocrinology and attention improvement during antidepressant treatment, as compared to those with normal BMI [34]. It can be hypothesized that the relationship between a high BMI and LOS may be attributed to the severity of the disease. Of note, antipsychotics and mood stabilizers are frequently prescribed together to severe psychiatric patients, which can result in significant weight gain [35]. Furthermore, inflammation may serve as a mediator in the relationship between elevated BMI and extended LOS. Inflammation is associated with both obesity and psychiatric disorders [36, 37], as well as disease severity [38], and may delay response to psychiatric treatments [39], likely leading to extended LOS. Of note, no significant association was found between LOS and BMI in FEP patients (i.e., patients with less severe disease and metabolic disturbances), which may be due, at least in part, to a limited sample size. Moreover, further research is needed to investigate the role of treatment-naïve versus long-term medicated patients in the development of metabolic disturbances and their subsequent impact on psychiatric illness and healthcare pathways, including hospital stays.

Central obesity was related to extended LOS, considering the whole cohort but not the FEP patients. Central adiposity is associated with stress, anxiety, inflammation, psychosis, and depression [40–42], which are prevalent among psychiatric patients. Additionally, this may be derived from the relationship between high BMI and extended LOS, as centrally obese patients had substantially higher BMI than non-centrally obese patients.

General population diabetic patients experience extended LOS in non-psychiatric hospitals as compared to non-diabetics [43]. Another general population study reported that hyperglycemia (glucose levels > 6.5 mmol/L) was associated with extended LOS in an acute medical unit [44]. The present study identified hyperglycemia as a predictor of extended LOS in psychiatric patients when considering the whole cohort, but not FEP patients. This could be attributed, in part, to the limited sample size of this subgroup, as well as the short duration of illness and exposure to psychotropic medications known for their metabolic risk profile, including hyperglycemia [45]. Further studies are needed to confirm this finding and provide a mechanistic explanation.

Patients with hypertriglyceridemia had a 13% and 18% increased risk of extended LOS when considering the entire cohort and patients experiencing their FEP, respectively. Interestingly, when only the initial hospital stay of the subgroup is considered, hypertriglyceridemia is the only metabolic disturbance that tends to be associated with extended LOS. The lack of statistical significance could be attributed, in part, to the small sample size (*n* = 130). One study found that lipoprotein levels are associated with antipsychotic treatment tolerance in patients with schizophrenia, which in turn may influence LOS [46]. We recently reported that hypertriglyceridemia is associated with a 21% increased risk of relapse and readmission to a psychiatric hospital [19]. Interestingly, elevated lipid levels are associated with smaller brain structures, more severe mood symptoms and cognitive dysfunction, poorer sleep quality, and increased impulsivity in bipolar patients, all of which can affect LOS [47, 48].

Contrary to the other metabolic disturbances, HDL hypocholesterolemia is the only one linked to shorter LOS in the entire cohort. However, patients experiencing their FEP did not exhibit this association. A study exploring depressive episodes in bipolar disorder patients found that elevated HDL cholesterol levels were associated with prolonged LOS [47]^.^ Interestingly, the severity of symptoms and other prognostic factors in individuals with bipolar disorders have been shown to be associated with lipid levels [47]. In a recent study, we did not observe any significant association between HDL hypocholesterolemia and readmission when considering the entire cohort; however, HDL hypocholesterolemia was associated with a twofold increase in the risk of readmission in FEP patients [19]. Further research is necessary to gain a deeper understanding of the relationship between HDL hypocholesterolemia and LOS in psychiatric hospitals, potentially focusing on the treatment response.

Hypertension showed no link with LOS in the whole cohort, while it was associated with an increased risk of staying longer in the psychiatric hospital in FEP patients. Given the sample size (*n* = 296), these results should be interpreted cautiously, and further research is necessary to confirm these findings.

Given the positive relationship between central obesity, hyperglycemia, hypertriglyceridemia, and the risk of extended LOS, it is not surprising to find the same relationship between the metabolic syndrome and extended LOS. Many factors can affect metabolic parameters, leading to the development of the metabolic syndrome in the psychiatric population, namely the psychiatric disorder itself [49], psychotropic drug use [14], poor diet and lifestyle [15], and genetics [18].

The effect of age on LOS in psychiatric hospitals is inconsistent between studies, depending on the modeling approaches used, with a possibility of a non-linear effect resulting in the shortest LOS in the middle age range [4, 10, 50]. Our study revealed extended LOS for those 65 years or older, which could be attributed to the complexity of managing such patients requiring multidisciplinary healthcare management.

The literature is unclear on the association between sex and LOS [10]. No relationship between sex and LOS was observed in the present study, potentially due, in part, to the distribution of psychiatric diagnoses between males and females. Indeed, females are more prone to depression [51], which is connected to shorter LOS in our study, whereas males are more prone to substance use disorders [52], likewise linked to shorter LOS.

In the present study, smokers were more likely to have shorter stays in the hospital, which could be due, in part, to the positive effect of nicotine on cognitive performance enhancement [53].

In terms of psychiatric diagnoses, our findings indicated that intellectual disabilities were associated with shorter LOS, which could be attributed to the department’s policy during the last decade of institutionalizing such patients in specialized settings.

Our results linked substance use disorders to shorter LOS, supporting prior findings [26, 54]. We recently found a significant association between substance use disorders and increased risk of readmission to a psychiatric hospital [19]. Lack of sustained motivation to remain abstinent often leads to premature release, relapse, and readmission.

Contrary to prior results, our study found no association between psychotic, schizoaffective, and bipolar disorders and LOS [6, 10, 54], which can be attributed to methodological aspects, namely diagnostic groups’ classification and the comparison group. Similarly, our results linked depressive disorders to shorter LOS, while previous studies showed no significant association with LOS. Indeed, the majority of studies considered depression and bipolar disorders together as mood or affective disorders, and/or considered a different comparator group [6, 50, 55].

A history of a previous hospital stay was linked to a greater likelihood of shorter LOS, which could tentatively be attributed to already-set-up procedures for outpatient follow-up, enabling an earlier discharge.

No significant association was found between weight-gain-inducing psychotropic medication and LOS. The moderate proportions of patients using psychotropic drugs known for their risk of weight gain (29%) compared to no risk (71%) may possibly account for the absence of such an association. Finally, corroborating a previous finding [6], our multivariable models indicate that LOS appears to decrease over time.

The present study had some limitations. Being an observational study, no causal link could be established. In addition, while our study examines the association between metabolic disturbances and LOS, we acknowledge that the relationship may be bi-directional. A hospital stay may induce metabolic alterations, including weight gain, attributable to reduced physical activity and modifications in psychiatric medication, namely those known for their risk of weight gain. Consequently, the observed associations should be interpreted with caution, and further research is needed to clarify the directionality of this relationship. The important number of missing data in some variables (e.g., admission status [voluntary or compulsory], education, employment, and living situation) previously associated with LOS did not allow adjusting for all the selected covariates in one single multivariate model. Furthermore, some previously identified predictors of LOS were not available in our database (e.g., the Clinical Global Impressions (CGI) scale, ethnicity, receiving electroconvulsive therapy, history of suicide attempts, and treatment setting after discharge). Moreover, models were unable to account for some potential confounding factors, including underlying psychiatric severity, lifestyle factors, and medication adherence. Finally, the cohort included predominantly Caucasian descent; future studies must validate these associations in other populations.

## Conclusion

The study of the association between metabolic disturbances and LOS in psychiatric patients, including FEP patients, is still preliminary, and further studies are needed to replicate the present findings. Efforts should be directed toward better metabolic monitoring, treatment of metabolic disturbances, and evaluation of their possible implications for reducing LOS over time.

## Supporting information

Laaboub et al. supplementary material Laaboub et al. supplementary material

## Data Availability

Due to the sensitive nature of the data and the lack of informed consent for public deposition, the datasets analyzed in this study are not publicly available. The data supporting the conclusions of this article were obtained from the PsyMetab study. Requests for access to the datasets can be directed to research.psymetab@chuv.ch.

## Supplementary material

The supplementary material for this article can be found at http://doi.org/10.1192/j.eurpsy.2025.10036.
