# Supplementary material for: Metabolic syndrome and its components are associated with lengths of stay in a psychiatric hospital: Results from a Swiss psychiatric cohort and first-episode psychosis patients
Source: Eur Psychiatry. 2025 May 26;68(1):e65. doi: 10.1192/j.eurpsy.2025.10036 (PMC12303775; doi:10.1192/j.eurpsy.2025.10036)
Supplement: Laaboub et al. supplementary material [file S0924933825100369sup001.docx]

**Metabolic syndrome and its components are associated with lengths of stay in a psychiatric hospital: results from a Swiss psychiatric cohort and first-episode psychosis patients: Supplementary files**

**Supplementary Table 1:** Psychotropic medications categorized by risk of weight gain

| **Risk of weight gain** | | **No risk of weight gain** |
| --- | --- | --- |
| Amisulpride | Lurasidone | All the other psychotropic medications |
| Amitriptyline | Mirtazapine |  |
| Aripiprazole | Nortriptyline |  |
| Asenapine | Olanzapine |  |
| Brexpiprazole | Opipramol |  |
| Carbamazepine | Pipamperone |  |
| Cariprazine | Pregabalin |  |
| Chlorprothixene | Promazine |  |
| Clomipramine | Quetiapine |  |
| Clotiapine | Risperidone/Paliperidone |  |
| Clozapine | Sertindole |  |
| Doxepin | Sulpiride |  |
| Flupentixol | Tiapride |  |
| Haloperidol | Trimipramine |  |
| Levomepromazine | Valproate |  |
| Lithium | Zuclopenthixol |  |

Psychotropic medications were categorized by their risk of weight according to:

Leucht S, Cipriani A, Spineli L, Mavridis D, Orey D, Richter F, et al. Comparative efficacy and tolerability of 15 antipsychotic drugs in schizophrenia: a multiple-treatments meta-analysis. Lancet. 2013;382:951–62.

Christoph U. Correll, Johan Detraux, Jan De Lepeleire, Marc De Hert. Effects of antipsychotics, antidepressants and mood stabilizers on risk for physical diseases in people with schizophrenia, depression and bipolar disorder. World Psychiatry. 2015

**Supplementary Table 2A:** Definition of metabolic disturbances and metabolic syndrome [International Diabetes Federation (IDF) definition]:

| A. Central obesity | Waist circumference ≥ 94 cm (male) or 80 cm (female) or BMI > 30 kg/m². |
| --- | --- |
| B. Hypertension | Systolic BP ≥ 130 or diastolic BP ≥ 85 mmHg and/or specific treatment for blood pressure abnormality is listed in Supplementary Table 2B. |
| C. Hyperglycemia | Fasting plasma glucose (FPG) ≥ 100 mg/dL (5.6 mmol/L) and/or specific treatment for type 2 diabetes is listed in Supplementary Table 2B. |
| D. Hypertriglyceridemia | Serum triglycerides ≥ 150 mg/dL (1.7 mmol/L) and/or treatment for lipid abnormality is listed in Supplementary Table 2B. |
| E. HDL hypocholesterolemia | HDL cholesterol < 40 mg/dL in men (1.03 mmol/L) or 50 mg/dL in women (1.29 mmol/L) and/or treatment for lipid abnormality is listed in Supplementary Table 2B. |
| Metabolic syndrome | Presence of A plus any two of B, C, D and E. |

BMI=body mass index; cm=centimeters; Kg/m^2^=kilograms per square meter; BP=blood pressure; mmHG=millimeters of mercury; mg/dL=milligram per deciliter; mmol/L=millimoles per liter; HDL=high-density lipoprotein.

**Supplementary Table 2B:** Treatment used to define hypertension (anti-hypertensive), hyperglycemia (anti-diabetic), and hypertriglyceridemia and HDL hypocholesterolemia (lipid lowering agents)

| **Lipid-lowering agents** | **Anti-diabetic medications** | **Antihypertensives** | | |
| --- | --- | --- | --- | --- |
| Simvastatin (C10AA01)  Pravastatin (C10AA03)  Fluvastatin (C10AA04)  Atorvastatin (C10AA05)  Rosuvastatin (C10AA07)  Pitavastatin (C10AA08)  Bezafibrate (C10AB02)  Gemfibrozil (C10AB04)  Fenofibrate (C10AB05)  Colestyramine (C10AC01)  Ezetimibe (C10AX09)  Simvastatin and ezetimibe (C10BA02) | Human Insulin (A10AB01, A10AC01, A10AD01)  Insulin lispro (A10AB04, A10AD04)  Insulin aspart (A10AB05, A10AD05)  Insulin glulisine (A10AB06)  Insulin degludec and insulin aspart (A10AD06)  Insulin glargine (A10AE04)  Insulin detemir (A10AE05)  Insulin degludec (A10AE06)  Metformin (A10BA02)  Glibenclamide (A10BB01)  Gliclazide (A10BB09)  Glimépiride (A10BB12)  Metformin and  sulfonamides (A10BD02)  rosiglitazone (A10BD03)  sitagliptin (A10BD07)  vildagliptin (A10BD08)  saxagliptin (A10BD10)  linagliptin (A10BD11)  empagliflozin (A10BD20)  Acarbose (A10BF01)  Rosiglitazone (A10BG02)  Pioglitazone (A10BG03)  Sitagliptin (A10BH01)  Vildagliptin (A10BH02)  Saxagliptin (A10BH03)  Linagliptin (A10BH05)  Exenatide (A10BJ01)  Liraglutide (A10BJ02)  Dapagliflozin (A10BK01)  Empagliflozin (A10BK03)  Repaglinide (A10BX02)  Nateglinide (A10BX03)  Desmopressin (H01BA02) | Clonidine (C02AC01)  Moxonidine (C02AC05)  Doxazosin (C02CA04)  Hydrochlorothiazide (C03AA03)  Chlortalidone (C03BA04)  Metolazone (C03BA08)  Indapamide (C03BA11)  Furosemide (C03CA01)  Torasemide (C03CA04)  Spironolactone (C03DA01)  Eplerenone (C03DA04)  Hydrochlorothiazide and potassium-sparing diuretics (C03EA01)  Butizide and potassium-sparing diuretics (C03EA14)  Furosemide and potassium-sparing diuretics (C03EB01)  Oxprenolol (C07AA02)  Propranolol (C07AA05)  Metoprolol (C07AB02)  Atenolol (C07AB03)  Bisoprolol (C07AB07)  Celiprolol (C07AB08)  Nebivolol (C07AB12)  Labetalol (C07AG01)  Carvedilol (C07AG02)  Bisoprolol and thiazides (C07BB07)  Nebivolol and thiazides (C07BB12)  Atenolol and other diuretics (C07CB03)  Metoprolol and other antihypertensives (C07FB02)  Atenolol and other antihypertensives (C07FB03) | Amlodipine (C08CA01)  Felodipine (C08CA02)  Isradipine (C08CA03)  Nifedipine (C08CA05)  Lercanidipine (C08CA13)  Verapamil (C08DA01)  Diltiazem (C08DB01)  Captopril (C09AA01)  Enalapril (C09AA02)  Lisinopril (C09AA03)  Perindopril (C09AA04)  Ramipril (C09AA05)  Quinapril (C09AA06)  Benazepril (C09AA07)  Cilazapril (C09AA08)  Fosinopril (C09AA09)  Captopril and diuretics (C09BA01)  Enalapril and diuretics (C09BA02)  Lisinopril and diuretics (C09BA03)  Perindopril and diuretics (C09BA04)  Ramipril and diuretics (C09BA05)  Quinapril and diuretics (C09BA06)  Cilazapril and diuretics (C09BA08)  Fosinopril and diuretics (C09BA09)  Perindopril and amlodipine (C09BB04)  Ramipril and calcium antagonists (C09BB05)  Trandolapril and calcium antagonists (C09BB10) | Losartan (C09CA01)  Valsartan (C09CA03)  Irbesartan (C09CA04)  Candesartan (C09CA06)  Telmisartan (C09CA07)  Olmesartan medoxomil (C09CA08)  Azilsartan medoxomil (C09CA09)  Losartan and diuretics (C09DA01)  Eprosartan and diuretics (C09DA02)  Valsartan and diuretics (C09DA03)  Irbesartan and diuretics (C09DA04)  Candesartan and diuretics (C09DA06)  Telmisartan and diuretics (C09DA07)  Olmesartan medoxomil with diuretics (C09DA08)  Azilsartan medoxomil and diuretics (C09DA09)  Valsartan and amlodipine (C09DB01)  Olmesartan medoxomil with amlodipine (C09DB02)  Valsartan, amlodipine with hydrochlorothiazide (C09DX01)  Olmesartan medoxomil, amlodipine with hydrochlorothiazide (C09DX03)  Valsartan and sacubitril (C09DX04)  Aliskiren (C09XA02)  Aliskiren and hydrochlorothiazide (C09XA52) |

Defined using: https://icd.who.int/, https://www.whocc.no/atc_ddd_index, and https://compendium.ch/

**Supplementary Table 3:** Evolution of Lengths Of Stay in days (LOS) over years in the whole cohort

| **Year** | **Mean** | **Standard deviation** | **Median** | **Quartile 1** | **Quartile 3** | **Number of observations*** |
| --- | --- | --- | --- | --- | --- | --- |
| **2007** | 37 | 57 | 19 | 8 | 41 | 1549 |
| **2008** | 38 | 52 | 20 | 9 | 45 | 1486 |
| **2009** | 39 | 56 | 22 | 10 | 45 | 1516 |
| **2010** | 35 | 44 | 20 | 9 | 41 | 1640 |
| **2011** | 38 | 54 | 21 | 9 | 46 | 1591 |
| **2012** | 42 | 58 | 23 | 11 | 51 | 1359 |
| **2013** | 39 | 49 | 24 | 12.5 | 45 | 1508 |
| **2014** | 40 | 68 | 24 | 13 | 45 | 1420 |
| **2015** | 35 | 46 | 23 | 13 | 40 | 1519 |
| **2016** | 42 | 56 | 25 | 14 | 47 | 641 |
| **2017** | 40 | 49 | 26 | 14 | 45 | 569 |
| **2018** | 41 | 50 | 28 | 15 | 48 | 591 |
| **2019** | 41 | 45 | 27 | 16 | 51 | 650 |
| **2020** | 32 | 29 | 23 | 13 | 42 | 811 |

* The decrease in the number of observations from 2016 onwards is attributed to the inclusion of data from the PsyMetab cohort only. Indeed, the PsyClin cohort only covers observations collected between 2007 and 2015.

**Supplementary Table 4:** Association between metabolic syndrome and Lengths Of Stay (LOS) in the whole cohort

| ***Predictors*** | ***Hazard Ratio*** | ***95% confidence interval*** | ***p-value*** |
| --- | --- | --- | --- |
| **Metabolic syndrome** (No: reference; n=4´157)  Yes (n=1´599) | 0.76 | 0.70 – 0.82 | **<0.001** |
| **Age** (18-25 years: reference; n=610)  25-39 years (n=1´881)  40-64 years (n=2´378)  65 years or older (n=887) | 1.12  1.02  0.60 | 0.99 – 1.26  0.90 – 1.16  0.51 – 0.70 | 0.08  0.76  **<0.001** |
| **Sex** (Male: reference; n=2´728)  Female (n=3´028) | 1.00 | 0.92 – 1.08 | 0.95 |
| **Smoking** (No: reference; n=2´222)  Yes (n=3´534) | 1.07 | 0.99 – 1.16 | 0.09 |
| **Psychiatric diagnosis** (others: reference; n=37)  Dementia (n=296)  Intellectual disabilities (n=144)  Substance use disorders (n=854)  Psychotic disorders (n=2´477)  Bipolar disorders (n=906)  Depression (n=1´042) | 0.68  1.42  1.20  0.83  0.83  1.20 | 0.46 – 1.02  0.90 – 2.23  0.80 – 1.79  0.56 – 1.23  0.56 – 2.24  0.81 – 1.79 | 0.06  0.13  0.38  0.36  0.36  0.36 |
| **Psychotropic medication** (No risk of metabolic disturbances: reference; n=4´095)  Risk of metabolic disturbances (n=1´661) | 1.01 | 0.94 – 1.08 | 0.82 |
| **Previous hospital admission** (No: reference; n=1´777) Yes (n=3´979) | 0.92 | 0.85 – 0.99 | **0.02** |
| **Year** (for each additional year) | 1.04 | 1.03 – 1.05 | **<0.001** |
| Observations | 5´756 | | |
| Variance | 41% | | |

Significant p-value in bold.

Psychiatric diagnoses were defined as follows: dementia [F00-F02 and G30], intellectual disabilities [F70-F79], substance use disorders [F10-F19], psychotic disorders [F20-F25 and F28-F29], bipolar disorders [F30-F31], depression [F32-F33], and other diagnoses [F03-F09, F34-F69, and F80-F99].

Metabolic syndrome defined using the International Diabetes Federation definition.

Psychotropic treatments were defined as follows: Risk of weight gain= amisulpride, amitriptyline, aripiprazole, asenapine, brexpiprazole, carbamazepine, cariprazine, chlorprothixene, clomipramine, clotiapine, clozapine, doxepin, flupentixol, haloperidol, levomepromazine, lithium, lurasidone, mirtazapine, nortriptyline , olanzapine, opipramol, pipamperone, pregabalin, promazine, quetiapine, risperidone/paliperidone, sertindole, sulpiride, tiapride, trimipramine, valproate, zuclopenthixol. No risk of weight gain= all the other psychotropic medications.

**Supplementary Table 5:** Association between metabolic syndrome and Lengths Of Stay (LOS) in the whole cohort considering time-varying effects (sensitivity analyses)

| ***Predictors*** | ***Hazard Ratio*** | ***95% confidence interval*** | ***p-value*** |
| --- | --- | --- | --- |
| **Metabolic syndrome** (No: reference; n=4´157)  Yes (n=1´599) | 0.76 | 0.71 – 0.82 | **<0.001** |
| **Age** (18-25 years: reference; n=610)  25-39 years (n=1´881)  40-64 years (n=2´378)  65 years or older (n=887) | 1.11  1.00  0.57 | 0.98 – 1.25  0.88 – 1.14  0.49 – 0.67 | 0.11  0.96  **<0.001** |
| **Sex** (reference male; n=2´728)  Female (n=3´028) | 1.00 | 0.93 – 1.08 | 0.92 |
| **Smoking** (No: reference; n=2´222)  Yes (n=3´534) | 1.15 | 1.05 – 1.26 | **0.003** |
| **Psychiatric diagnosis** (others: reference; n=37)  Dementia (n=296)  Intellectual disabilities (n=144)  Substance use disorders (n=854)  Psychotic disorders (n=2´477)  Bipolar disorders (n=906)  Depression (n=1´042) | 0.91  3.05  2.55  1.34  1.15  2.14 | 0.46 – 1.79  1.50 – 6.20  1.32 – 4.92  0.70 – 2.58  0.59 – 2.23  1.11 – 4.13 | 0.78  **0.002**  **0.005**  0.38  0.68  **0.02** |
| **Psychotropic medication** (reference: no risk of metabolic disturbances; n=4´095)  Risk of metabolic disturbances (n=1´661) | 1.01 | 0.95 – 1.08 | 0.74 |
| **Previous hospital admission** (reference: no; n=1´777)  Yes (n=3´979) | 1.11 | 1.01 – 1.22 | **0.03** |
| **Year** (for each additional year) | 1.04 | 1.03 – 1.06 | **<0.001** |
| Length of stay (LOS)× Smoking (No) | 1.094 | 1.093 – 1.096 | **<0.001** |
| Length of stay (LOS) × Diagnosis (dementia)  Length of stay (LOS) × Diagnosis (intellectual disabilities)  Length of stay (LOS) × Diagnosis (substance use disorders)  Length of stay (LOS) × Diagnosis (psychotic)  Length of stay (LOS) × Diagnosis (bipolar)  Length of stay (LOS) × Diagnosis (depression) | 0.992  0.982  0.982  0.990  0.994  0.988 | 0.982 – 1.002  0.970 – 0.993  0.973 – 0.992  0.981 – 1.000  0.984 – 1.004  0.978 – 0.998 | 0.11  **0.001**  **0.0004**  **0.049**  0.24  **0.02** |
| Length of stay (LOS) × Previous hospital admission (Yes) | 0.995 | 0.993 – 0.996 | **<0.001** |
| Observations | 5´756 | | |

Significant p-value in bold.

Psychiatric diagnoses were defined as follows: dementia [F00-F02 and G30], intellectual disabilities [F70-F79], substance use disorders [F10-F19], psychotic disorders [F20-F25 and F28-F29], bipolar disorders [F30-F31], depression [F32-F33], and other diagnoses [F03-F09, F34-F69, and F80-F99].

Metabolic syndrome was defined using the International Diabetes Federation definition.

Psychotropic treatments were defined as follows: Risk of weight gain= amisulpride, amitriptyline, aripiprazole, asenapine, brexpiprazole, carbamazepine, cariprazine, chlorprothixene, clomipramine, clotiapine, clozapine, doxepin, flupentixol, haloperidol, levomepromazine, lithium, lurasidone, mirtazapine, nortriptyline , olanzapine, opipramol, pipamperone, pregabalin, promazine, quetiapine, risperidone/paliperidone, sertindole, sulpiride, tiapride, trimipramine, valproate, zuclopenthixol. No risk of weight gain= all the other psychotropic medications.

**Supplementary Table 6**: Socio-demographic and clinical characteristics of First Episode of Psychosis patients

| ***Characteristics*** | ***Total of admissions N=1’134*** | ***Characteristics*** | ***Total of admissions N=1’134*** |
| --- | --- | --- | --- |
| **LOS**, median (IQR), days | 25 (13-47) | **HoNOS score at admission**, median (IQR) | 21 (15-27) |
| **Age**, median (IQR), years | 27 (24-32) | **SSEP** median (IQR) | 54 (40-63) |
| **Sex**, n (%)  Men  Women | 674 (59)  460 (41) | **Smokers**, n (%)  No  Yes | 190 (28)  499 (72) |
| **Marital status**, n (%)  Married or registered partnership  Single  Divorced or separated  Widowed | 75 (7)  1’002 (88)  48 (4)  9 (1) | **Education**, n (%)  Compulsory schooling  No schooling  Apprenticeship  High school  University or college | 83 (39)  11 (5)  62 (29)  18 (9)  37 (18) |
| **Employment**, n (%)  Employed  Unemployed  Pensions [disability, retirement]  Other [e.g., student] | 31 (12)  75 (28)  136 (51)  23 (9) | **Admission status**, n (%)  Voluntary  Compulsory | 150 (49)  158 (51) |
| **Living situation**, n (%)  Home, with others  Home, alone  Homeless  Institutions  Others | 107 (36)  99 (33)  11 (4)  65 (22)  17 (5) | **Psychotropic treatments by risk of weight gain**, n (%)  No risk of weight gain  Risk of weight gain | 730 (64)  404 (36) |

N varies due to missing values

Psychotropic treatments were defined as follow: Risk of weight gain= amisulpride, amitriptyline, aripiprazole, asenapine, brexpiprazole, carbamazepine, cariprazine, chlorprothixene, clomipramine, clotiapine, clozapine, doxepin, flupentixol, haloperidol, levomepromazine, lithium, lurasidone, mirtazapine, nortriptyline , olanzapine, opipramol, pipamperone, pregabalin, promazine, quetiapine, risperidone/paliperidone, sertindole, sulpiride, tiapride, trimipramine, valproate, zuclopenthixol. No risk of weight gain= all the other psychotropic medications

Abbreviations: HoNOS=Health of the Nation Outcome Scales; IQR=interquartile range; LOS=length of stays; n=number; SSEP=Swiss socioeconomic position.

**Supplementary Table 7.** Metabolic characteristics of First Episode of Psychosis patients at admission

| ***Characteristics*** | ***Total observations N=1’134*** |
| --- | --- |
|  |  |
| **Weight**, median (IQR), kg | 75 (64-86) |
| **BMI**, median (IQR), kg/m^2^ | 24.5 (21.6-28.1) |
| **BMI categories**, n (%)   - Normal (18.5 kg/m^2^ ≤ BMI < 25 kg/m^2^) - Underweight (BMI < 18.5 kg/m^2^) - Overweight (25 kg/m^2^ ≤ BMI < 30 kg/m^2^) - Obese (BMI ≥ 30 kg/m^2^) | 304 (46)  52 (8)  192 (29)  113 (17) |
| **Waist circumference**, median (IQR), cm   - Men - Women | 87 (80-100)  91 (80-103) |
| 1. **Central obesity**, n (%), IDF definition  - Yes - No | 185 (27)  489 (73) |
| **Plasma cholesterol**   - Triglycerides, median (IQR), mmol/L - HDL, median (IQR), mmol/L   - Men   - Women - LDL, median (IQR), mmol/L - Total, median (IQR), mmol/L | 1.2 (0.9-1.7)  1.2 (1.1-1.5)  1.3 (1.1-1.7)  2.5 (2.0-3.1)  4.4 (3.8-5.1) |
| 1. **Hypertriglyceridemia**, n (%)  - Yes - No | 208 (29)  513 (71) |
| 1. **HDL hypocholesterolemia**, n (%)  - Yes - No | 284 (37)  487 (63) |
| **Systolic blood pressure**, median (IQR), mmHg | 129 (120-142) |
| **Diastolic blood pressure**, median (IQR), mmHg | 87 (80-95) |
| 1. **Hypertension**, n (%)  - Yes - No | 256 (72)  102 (28) |
| **Fasting plasma glucose**, median (IQR), mmol/L | 5.0 (4.6-5.4) |
| 1. **Hyperglycemia**, n (%)  - Yes - No | 150 (18)  674 (82) |
| 1. **Metabolic syndrome**, n (%), IDF definition  - Yes - No | 145 (19)  619 (81) |

N varies due to missing values

Abbreviations: BMI=Body Mass Index; cm=centimeter; HDL= High-Density Lipoprotein; IDF=International Diabetes Federation; IQR=interquartile range; kg=kilogram; L=liter; LDL=Low-Density Lipoprotein; m=meter; mmHg= millimeters of mercury; mmol=millimole, n= number of observations.

**Supplementary Table 8:** Evolution of Lengths Of Stay in days (LOS) over years in First Episode of Psychosis patients

| **Year** | **Mean** | **Standard deviation** | **Median** | **Quartile 1** | **Quartile 3** |
| --- | --- | --- | --- | --- | --- |
| **2007** | 31 | 34 | 21 | 10 | 37 |
| **2008** | 31 | 28 | 21 | 12 | 42 |
| **2009** | 31 | 39 | 21 | 10 | 39 |
| **2010** | 32 | 31 | 23 | 12 | 40 |
| **2011** | 40 | 51 | 21 | 10 | 51 |
| **2012** | 38 | 33 | 34 | 17 | 53 |
| **2013** | 32 | 36 | 22 | 10 | 41 |
| **2014** | 37 | 32 | 29 | 11 | 54 |
| **2015** | 42 | 53 | 33 | 17 | 48 |
| **2016** | 33 | 33 | 22 | 12 | 45 |
| **2017** | 35 | 32 | 26 | 18 | 44 |
| **2018** | 47 | 45 | 41 | 17 | 64 |
| **2019** | 42 | 35 | 33 | 21 | 55 |
| **2020** | 41 | 33 | 31 | 16 | 52 |

**Supplementary Figure 1:** Association between metabolic syndrome and Lengths Of Stay (LOS) in the whole cohort considering the time-varying effects (sensitivity analyses)

**
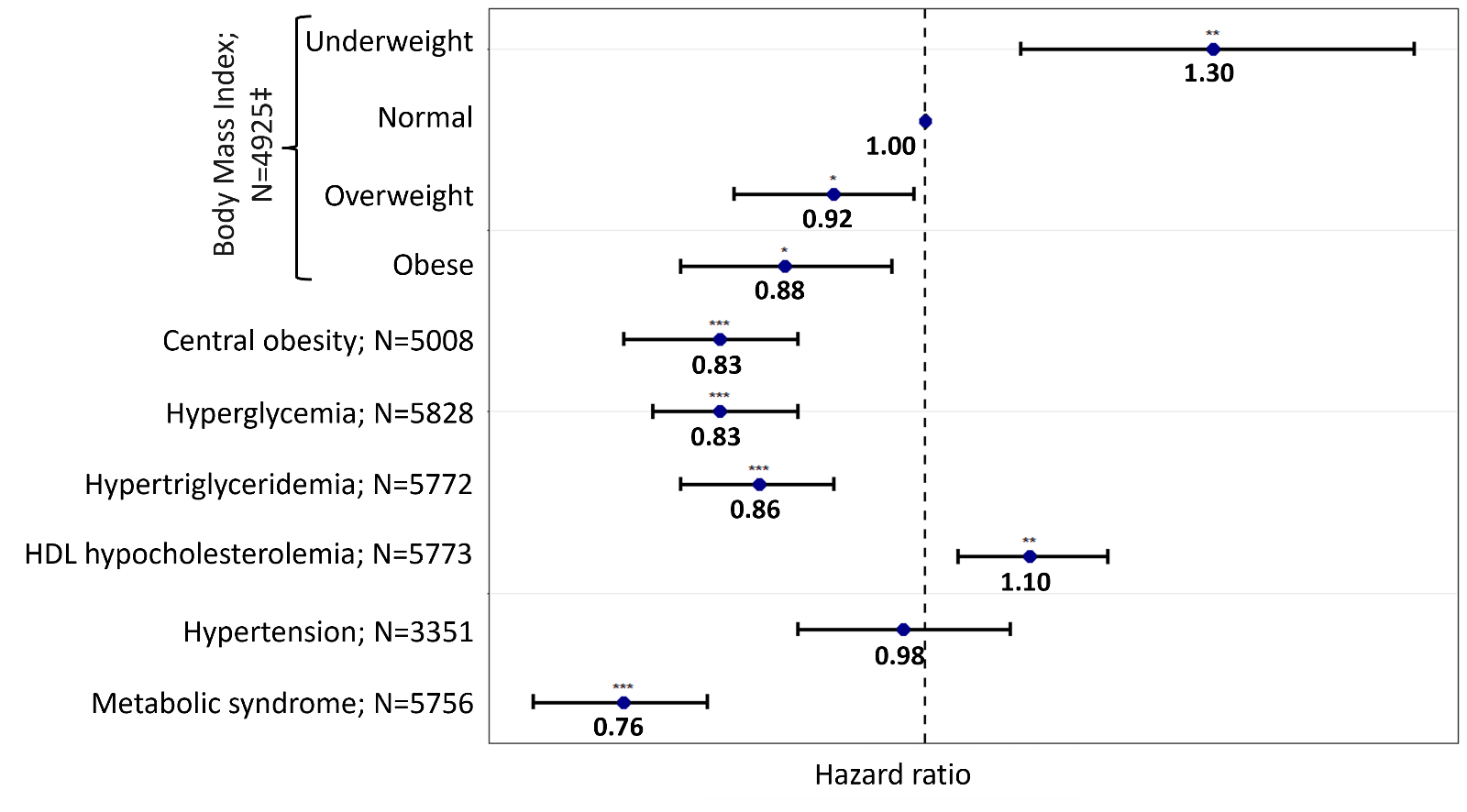
**

*: p-value<0.05; **: p-value<0.01; ***: p-value<0.001.

‡ Compared to normal weight and defined as follows: Normal weight (reference): 18.5≤ Body Mass Index (BMI) ≤ 25; Underweight: BMI <18.5 kg.m-2; Overweight: 25 ≤ BMI ≤ 30; Obese: BMI>30.

Each model was adjusted for age, sex, smoking status, psychiatric diagnoses, psychotropic medication, previous hospital admission, year, and interaction between time and smoking, psychiatric diagnoses, and previous hospital admission variables.

Abbreviation: HDL= high-density lipoprotein; N: number of observations in each model.

N varies due to missing values.

**Supplementary Figure 2:** Association between metabolic disturbances and Lengths Of Stay (LOS) in First Episode of Psychosis patients considering time-varying effects (sensitivity analyses):


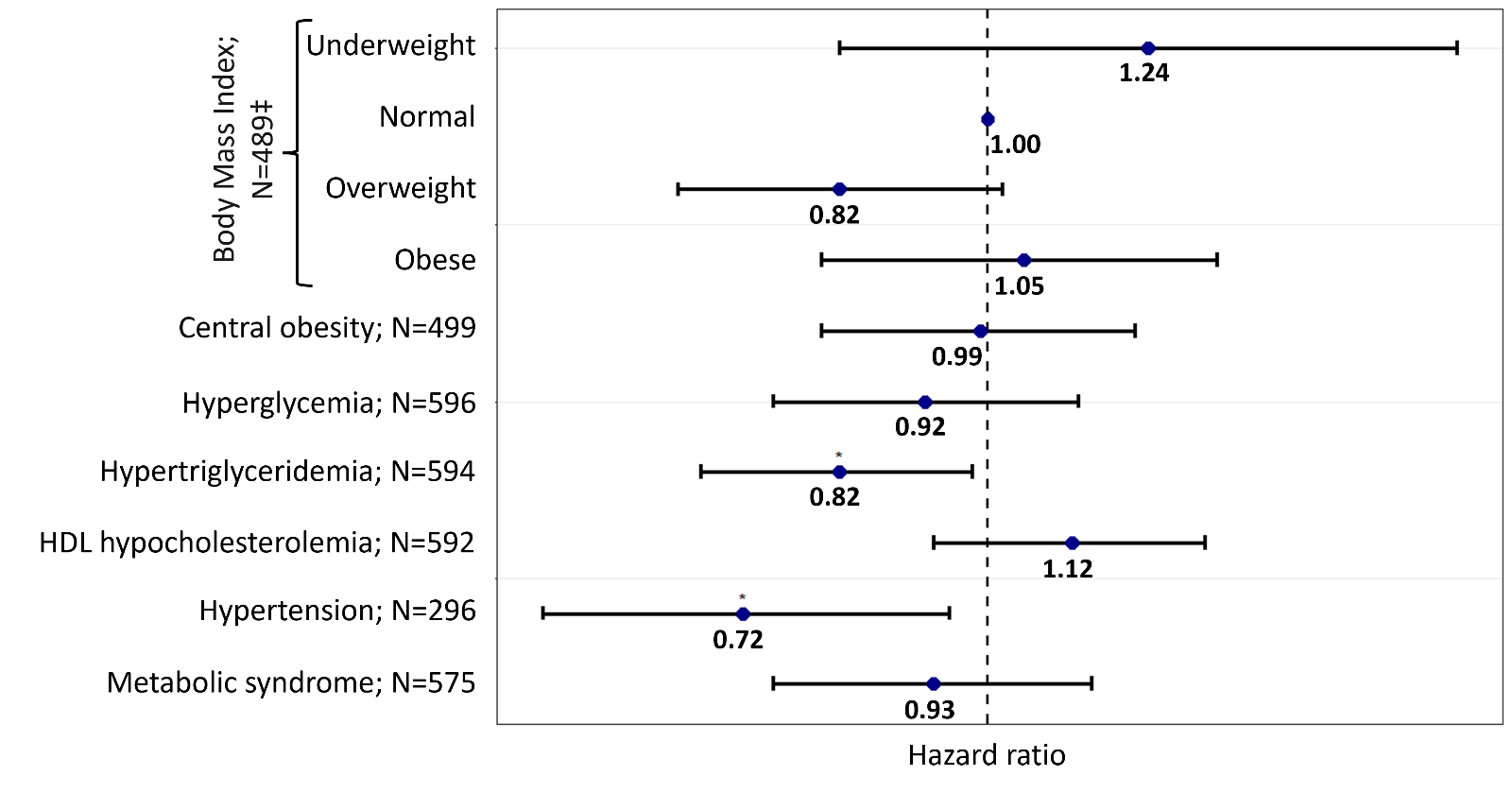


*: p-value<0.05.

N varies due to missing values.

‡Compared to normal weight and defined according to the World Health Organization (WHO) definition: Normal weight (reference): 18.5 kg.m^-2^≤ Body Mass Index (BMI) ≤ 25 kg.m^-2^; Underweight: BMI <18.5 kg.m^-2^; Overweight: 25 kg.m^-2^≤ BMI ≤ 30 kg.m^-2^; Obese: BMI>30 kg.m^-2^.

Metabolic disturbances defined using the International Diabetes Federation definition.

Models were adjusted for age, sex, smoking status, psychotropic medication, the year, and interaction between time and previous hospital admission.

Abbreviations: N: number of observations in the model.

**Supplementary Figure 3:** Association between metabolic disturbances and Lengths Of Stay (LOS) in First Episode of Psychosis patients (first hospital stay only):


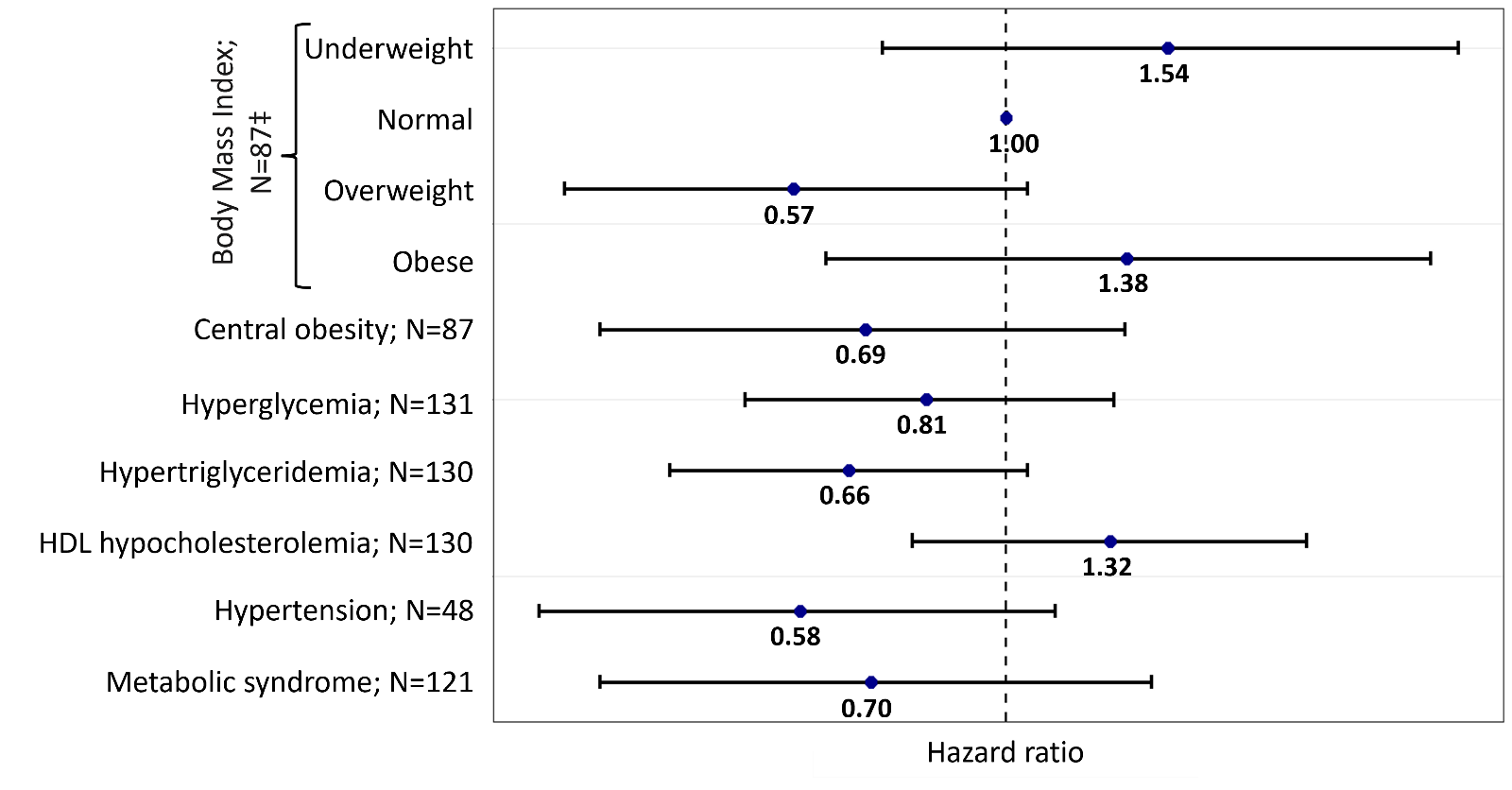


*: p-value<0.05.

N varies due to missing values.

‡Compared to normal weight and defined according to the World Health Organization (WHO) definition: Normal weight (reference): 18.5 kg.m^-2^≤ Body Mass Index (BMI) ≤ 25 kg.m^-2^; Underweight: BMI <18.5 kg.m^-2^; Overweight: 25 kg.m^-2^≤ BMI ≤ 30 kg.m^-2^; Obese: BMI>30 kg.m^-2^.

Metabolic disturbances defined using the International Diabetes Federation definition.

Models were adjusted for age, sex, smoking status, psychotropic medication, and the year.

Abbreviations: N: number of observations in the model.
